# CDK5 neutralizes the tumor suppressing effect of BIN1 via mediating phosphorylation of c-MYC at Ser-62 site in NSCLC

**DOI:** 10.1186/s12935-019-0952-5

**Published:** 2019-09-02

**Authors:** Xiangyu Zhang, Jiali Wang, Yunlong Jia, Tianxu Liu, Mengjie Wang, Wei Lv, Rong Zhang, Juan Shi, Lihua Liu

**Affiliations:** 1 Department of Tumor Immunotherapy, Fourth Hospital of Hebei Medical University and Hebei Cancer Institute, Tianshan Street 169, Shijiazhuang, China; 2 Department of Toxicology, Hebei Medical University, Shijiazhuang, China; 3 State Key Laboratory of Molecular Biology, Institute of Basic Medical Sciences, Chinese Academy of Medical Sciences & Peking Union Medical College, Beijing, China

**Keywords:** CDK5, BIN1, c-MYC, NSCLC

## Abstract

**Background:**

Bridging integrator 1 (BIN1) has showed outstanding tumor-suppressive potential via inhibiting c-MYC-mediated tumorigenesis. However, a frequent phosphorylation of c-MYC at Ser-62 site could block the BIN1/c-MYC interaction and limits the tumor-suppressive effect of BIN1. Cyclin-dependent kinase 5 (CDK5), a generally dysregulated protein in various carcinomas, can mediate c-MYC phosphorylation at Ser-62 site. However, whether the existence of CDK5 could block the BIN1/c-MYC interaction remains unclear.

**Materials and methods:**

The expression of CDK5 and BIN1 in non-small cell lung cancer (NSCLC) cell lines were measured. CDK5 was knocked down and overexpressed in H460 and PC9 cells, respectively. CCK-8, wound healing and transwell were used to detect the proliferation, migration and invasion ability of NSCLC cells. Tumor-bearing nude mouse model was built with H460 cells. Dinaciclib was added to realize the effect of CDK5 inhibition in vivo. NSCLC and matched para-carcinoma specimens were collected from 153 patients who underwent radical operation. IHC was performed to determine the expression of CDK5 in the specimens. Kaplan–Meier analysis was used to analyze the correlation between the postoperative survival and CDK5 expression.

**Results:**

CDK5 was highly expressed in H460 cells, and knockdown of CDK5 could restore the BIN1/c-MYC interaction. Meanwhile, low expression of CDK5 was observed in PC9 cells, and overexpression of CDK5 blocked the BIN1/c-MYC interaction. Consequently, the growth, migration, invasion and epithelial mesenchymal transition (EMT) ability of H460 and PC9 cells could be facilitated by CDK5. The addition of CDK5 inhibitor Dinaciclib significantly suppressed the tumorigenesis ability of NSCLC cells in tumor-bearing mouse model. Furthermore, high expression of CDK5, along with low expression of BIN1, could predict poor postoperative prognosis of NSCLC patients. The patients with high expression of CDK5 and low expression of BIN1 showed similar prognosis, indicating that CDK5 could neutralize the tumor suppressing effect of BIN1 in clinical situation.

**Conclusions:**

CDK5 blocked the interaction of BIN1 and c-MYC via promoting phosphorylation of c-MYC at ser-62 site, ultimately facilitated the progression of NSCLC.

## Background

Non-small cell lung cancer (NSCLC) is one of the most common malignant tumor with high mortality rate worldwide [1]. Despite the current therapies including surgery, radiotherapy, chemotherapy and immunotherapy has notably improved patient survival, the overall survival of NSCLC patients remains unsatisfactory [2]. Insufficient understanding of related molecular mechanisms limits the improvement in NSCLC patient prognosis. c-MYC, a key transcription factor by binding on enhancer box sequences (E-boxes), is frequently dysregulated and acts as a tumor promoting protein in numerous cancers [3, 4]. c-MYC is essential for normal cell proliferation to occur, and its dysregulation could trigger cell malignant transformation and finally lead to carcinogenesis [5]. The aberrant activation of c-MYC can immortalize cells, facilitate cell cycle progression and suppress differentiation [6]. c-MYC is observed to be frequently amplified in NSCLC, promoting various kinds of tumor malignant behaviors such as proliferation, invasion, chemotherapy resistance and immune escape [7]. Importantly, phosphorylation of c-MYC on Ser-62 is indispensable for its malignant behaviors [8]. This phosphorylation site exerts opposing control of c-MYC degradation through the ubiquitin–proteasome pathway. In response to a growth-stimulatory signal, transcription of the c-MYC gene is increased and newly synthesized c-MYC protein is phosphorylated on the Ser-62 residue, which results in its stabilization [9].

Bridging integrator 1 (BIN1), also known as Myc box-dependent-interacting protein 1, was identified as a tumor suppressor interacting with MYC box 1, a highly conversed region of the c-MYC N terminus [10]. By binding with c-MYC, BIN1 could significantly inhibit proliferation and apoptosis ability while induce apoptosis of cancer cells [11, 12]. Our previous study has revealed that BIN1 could inhibit programmed death ligand 1 (PD-L1) mediated immune suppression by neutralizing the c-MYC induced PD-L1 upregulation [7]. A structural analysis showed a canonical interaction between the SH3 domain and the proline-rich region of c-MYC centered on two Xxx-Pro di-peptides P59-P60 and S62-P63. While the affinity of unphosphorylated Myc-55-68 for BIN1-SH3 was significant, and the peptide phosphorylated on Ser-62 was unable to bind BIN1-SH3 even at micromole concentrations [13]. Thus, the presence of the factors inducing phosphorylation of c-MYC on Ser-62 could deactivate the tumor suppressing effect of BIN1.

Cyclin-dependent kinase 5 (CDK5) is a proline-directed serine/threonine kinase that functions as tumor promoter in the development and progression of multiple cancers by regulating cell proliferation, apoptosis, DNA repair and immune escape [14–16]. Various researches have revealed that overexpression of CDK5 was associated with cancer progression and worse prognosis, and inhibition of CDK5 activity was determined to suppress growth in numerous cancers [17, 18]. It is noteworthy that CDK5 is identified to directly phosphorylate c-MYC on Ser-62 [19]. Taken above, aberrant activation of CDK5 might be able to limit the tumor suppressing effect of BIN1 by blocking the BIN1/c-MYC interaction, consequently, overcoming the overexpression of CDK5 might further improve the prognosis of NSCLC patients.

Here, we showed that the high expression of CDK5 neutralized the tumor suppressing effect of BIN1 by phosphorylating c-MYC at Ser-62 site. In addition, knockdown of CDK5 inhibited clone formation, proliferation, invasion and migration ability of NSCLC cells by restoring BIN1/c-MYC interaction. Meanwhile, CDK5 inhibitor Dinaciclib restored the function of BIN1 to suppress cell proliferation via interrupting phosphorylation of c-MYC at Ser-62 site. Furthermore, high expression of CDK5 along with low expression of BIN1 associated with worse prognosis of NSCLC patients, indicating a novel role of CDK5 in NSCLC.

## Materials and methods

### Patients and specimens

The specimens of NSCLC tissues and matched para-carcinoma tissues were collected from 153 patients who underwent radical operation at the Fourth Hospital of Hebei Medical University (Shijiazhuang, China) between Jan. 2015 and Feb. 2016. The median patient age at the time of surgery was 54 years (range: 25–72 years). None of the NSCLC patients received preoperative radiotherapy, chemotherapy and immunotherapy. The clinical stage and histological tumor type were determined according to the Uion of International Cancer Control (UICC) Classification of 2017 (8th edition). Patient’s clinical information, such as gender, age, TNM stage, invasion range, lymph node metastasis, was collected and stored in a database. All participant information was updated every 3 months by telephone follow-up. Complete follow-up was updated until death or Mar. 2019. Carcinoma tissue and para-carcinoma tissue specimens were collected and treated promptly after the surgery. The data of this research was approved for use by the ethic committee of the Hebei Medical University (approval number: 2018MEC065) and all informed consents were signed by patients.

### Reagents and materials

Antibodies to CDK5 (EP715), c-MYC (ab32072), phosphorylated c-MYC at Ser-62 (ab185656), GAPDH (ab9485) were purchased from Abcam. (Cambridge, MA, US). Antibodies to CDK5/p35 (PR4026C) was purchased from Thermo Fisher (Carlsbad, CA, US). The CDK5-expressing lentivirus vector pCDH-CDK5 (pCDH-CMV-MCS-EF1-copGFP) and the control vector pCDH (pCDH-CMV-MCS-EF1-puro) and CDK5-siRNA were purchased from Gene Pharma Company (Shanghai, China). GoTaq qPCR Master Mix was purchased from Promega (Madison, WI, USA). Revert Aid First Strand cDNA Synthesis Kits was purchased from MBI Fermentas (Hanover, MD, USA). CDK5 inhibitor Dinaciclib (sc-364483A) was purchased from Santa Cruz Biotechnology (Dallas, TX).

### RNA extraction and quantitative reverse-transcriptase PCR

Reverse-transcriptase PCR primers for human CDK5 mRNA on the basis of the sequence in NCBI (accession number U68485). CDK5: forward: 5′-GTCCATCGACATGTGGTCAG-3′; reverse: 5′-CTGGTCATCCACATCATTGC-3′, GAPDH forward: 5′-ACCACAGTCCATGCCATCACT-3′, reverse: 5′-TCCACCACCCTGTTGCTGTA-3′. The experiments were triplicated. The comparative threshold cycle (Ct) method was used to calculate the relative expression. The amount of target relative to a calibrator is given by 2^−∆∆Ct^ [∆Ct = Ct (target gene) − Ct (*GAPDH*), ∆∆Ct = ∆Ct (carcinoma specimen) − ∆Ct (matched para-carcinoma specimen)].

### Western blotting

Cells were lysed in lysis buffer, and protein concentrations were measured with the BCA protein assay kit (ThermoFisher). Proteins were separated by 10% SDS-PAGE and transferred electrophoreticly onto polyvinylidene difluoride membranes (Millipore, Billerica, MA, USA). The membranes were incubated in PBS containing 5% bovine serum albumin for 2 h at room temperature, followed by overnight incubation at 4 °C with different dilutions of the primary antibodies, including antibodies to CDK5, c-MYC, p-c-MYC (Ser-62). The membranes were developed with the Odyssey infrared imaging system according to the manufacturer’s instructions. The levels of protein in each sample were normalized relative to those of GAPDH. Each experiment contained triplicate wells of each sample, and all experiments were repeated at least three times.

### Expression plasmid construction and transient transfections

A eukaryotic expression plasmid of the human CDK5 gene was constructed using a pCDH vector (Invitrogen, China). We constructed CDK5 cDNA expression vector on the basis of the sequence in NCBI. The empty vector was used as negative control. PC9 cells were cultured in six-well plates until they reached 80–90% confluence, and then transient transfections were performed using Lipofectamine 2000 (Invitrogen, China) according to the manufacturer instructions. At 48 h after transfection, gene expression was confirmed via immunoblotting analysis and qRT-PCR.

### Knockdown of CDK5 with siRNA

To further investigate the mechanisms of immunomodulation by CDK5, the CDK5 gene was silenced with siRNA. The siRNA sequences were as follows: CDK5-siRNA-1: 5′-GCGUCCAGAAUUUCAACAATT-3′; CDK5-siRNA-2: 5′-CCACUACGAGUCCCUUCAATT-3′; CDK5-siRNA-3: 5′-GCGUAGGUUUCUACGUCAATT-3′; negative control: 5′-UUCUCCGAACGUGUCACGUTT-3′. The siRNAs were synthesized by GenePharma Company (Shanghai, China). A total of 400 pmol siRNA was transfected into 4 × 10^5^ H460 cells using Lipofectamine RNAi MAX reagent (Invitrogen, Grand Island, NY, USA) according to the manufacturer’s protocol.

### Co-immunoprecipitation

In order to obtain total protein, transfected cells were harvested and lysed using lysis buffer containing protease inhibitors. Co-immunoprecipitation (Co-IP) assay was performed using a Co-IP kit (Pierce, Rockford, USA) according to the manufacturer’s instructions. Briefly, 3 mg of total protein for each treatment was used in this study, pre-cleared with Sepharose resins. The supernatants were divided equally into 2 tubes and incubated into columns containing 1 µg immobilized anti-c-MYC or anti-BIN1 antibody (Abcam). The immunocomplexes were covalently associated with Sepharose resins. The resins were eluted 5 times and the co-immunoprecipitate were separated on an SDS-PAGE gel before transferring to a PVDF membrane for analysis with anti-c-MYC or anti-BIN1 antibodies.

### Cell proliferation assay

The proliferation of cells was measured by cell-counting kit-8 (CCK-8) assay according to the manufacturer’s protocol. Briefly, approximately 2 × 10^3^ cells were plated into 96-well plates. When cells adhered, 10 μl of CCK8 (Solarbio, Beijing, China) was added to each well and incubated for 2 h in a humidified incubator containing 5% CO_2_ at 37 °C. The absorbance of each well was detected at a wavelength of 450 nm. Proliferation rates were determined at 0, 24, 48, 72, 96 h after transfection. Experiments were performed in triplicate.

### Wound-healing experiments

5 × 10^5^ H460 and PC9 cells were seeded in 24-well plates. After scraping the cell monolayer with a sterile micropipette tip, the wells were washed with serum-free medium in triplicate. The first image of each scratch was acquired at time zero. After 24 h, each scratch was examined and captured at the same location. Then, the healed area was measured for calculating the index of cell migration.

### Transwell invasion assay

Tumor cell migration assay was performed in a 24-well transwell chamber (Corning, NY, USA), which contained an 8 μm pore size polycarbonate membrane filter and was precoated with 100 μg Matrigel for invasion assay (Becton–Dickinson, Bedford, USA). Briefly, the cells were seeded in the upper chambers and incubated in 500 μl RPMI 1640 medium without FBS, while 500 μl medium with 10% FBS was placed in the lower chambers. The plates were incubated for 24 h in a 5% CO_2_ humidified incubator at 37 °C. Cells on the upper side of the filters were removed by cotton-tipped swabs, and the filters were washed with PBS. Then the cells on the lower side were fixed in 4% formaldehyde and stained with 1% crystal violet in PBS for 5 min at room temperature. The cells on the lower side of the filters were defined as migration cells and counted at 200× magnification in 5 random fields of each filter.

### Treatment with Dinaciclib

NSCLC cell lines H460 and PC9 were grown in RPMI supplemented with 10% FBS. A stock solution of Dinaciclib (1 nM) or vehicle control-DMSO was diluted in fresh tumor medium and added to samples to achieve a final concentration of 10 μM or 20 μM. After 4 days of treatment for 24 h and then harvested for cell proliferation analysis.

### In vivo tumor growth assay

BALB/c nude mice were used for tumor growth assay in vivo. Care was performed according to the National Research Council Guide for the Care and Use of Laboratory Animals, and was approved by the Institutional Animal Care and Use Committee (IACUC) of Hebei Medical University, Shijiazhuang, China. Tumor cells were harvested with trypsin solution and resuspended in PBS. Cells (1 × 10^6^ cells/mouse) in 0.1 ml were injected subcutaneously into BALB/c nude mice. The mice were divided randomly to 2 groups: Dinaciclib treated group and control group. All mice were sacrificed by cervical dislocation on the day 32.

### Immunohistochemistry assay

Immunohistochemistry (IHC) analysis was performed as we previously described [20]. The rabbit polyclonal antibody against CDK5 was used for detection. For evaluating expression of CDK5 in NSCLC tissues, the staining was visualized and classified based on the percentage of positive cells and the intensity of staining according to the 0–4 semi-quantitative system described in our previous study [20]. The total scores were determined by multiplying the percentage score and intensity score and graded as low for score of 0–4 and high for score of 5–12. Each section was scored independently by two pathologists and a third pathologist determined the final score if there was any inconsistency.

### Immunofluorescence

PC9 and H460 cells harvested and incubated with rabbit to human BIN1, mouse to human CDK5 mAb at 4 °C overnight. The cells were then stained by FITC conjugated goat anti-mouse and PE conjugated goat anti-rabbit antibody, followed by DAPI staining of the nucleus. The fluorescence was observed and analyzed with a fluorescence microscope at high magnification (200×).

### Statistical analysis

Statistical analysis was performed by using SPSS statistics software, version 25.0 (SPSS, Chicago, IL, USA). Spearman rank correlation was used to analyze the association of CDK5 and BIN1 expression in NSCLC specimens. Survival analysis was carried out using the log-rank test in association with Kaplan–Meier analysis and Cox proportional hazards model analysis. A P value < 0.05 was considered to be statistically significant, and all P values were two-tailed.

## Results

### CDK5 blocked the BIN1/c-MYC interaction via mediating phosphorylation of c-MYC on Ser-62 in NSCLC cells

**Fig. 1:**
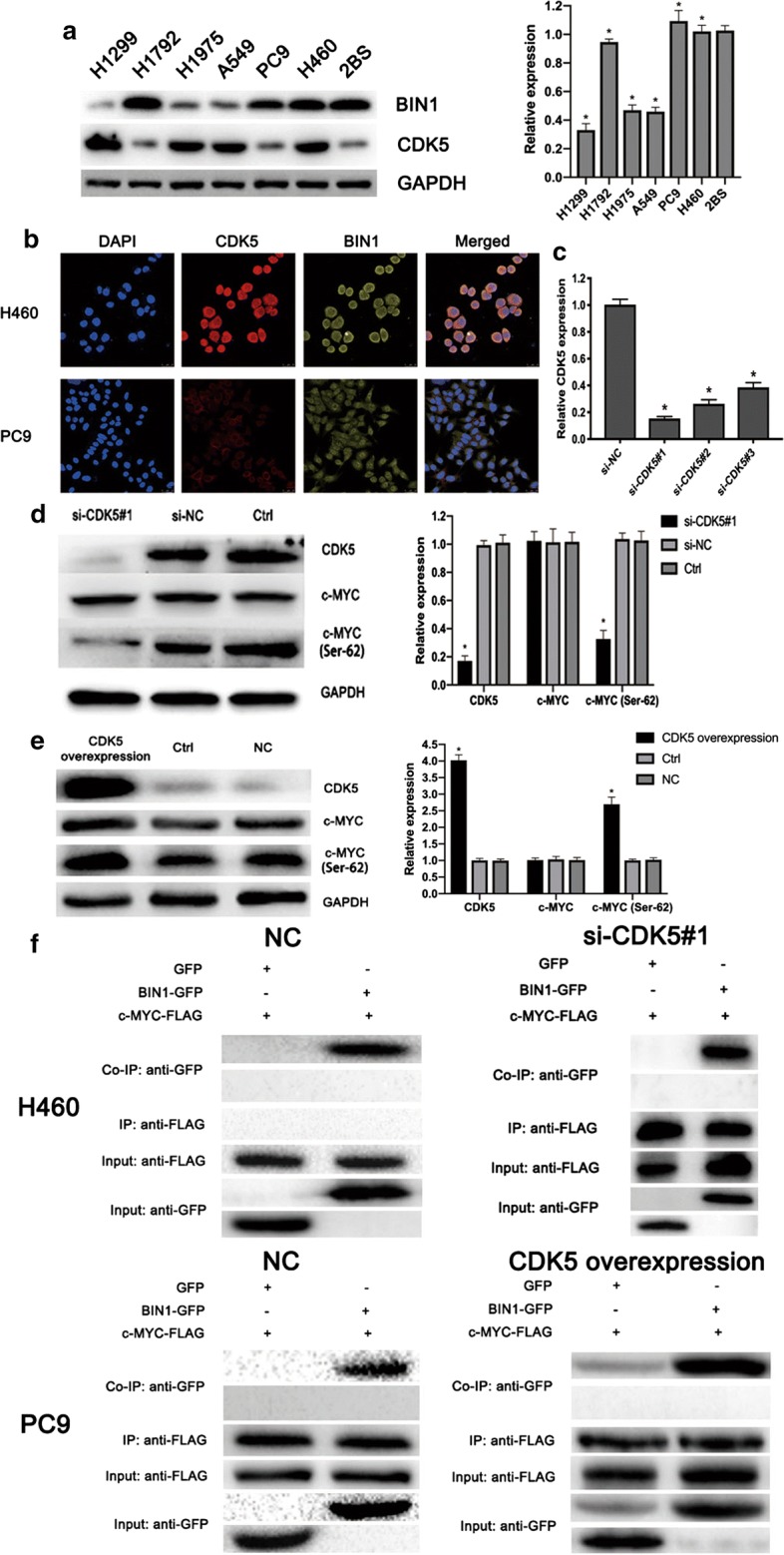
The expression of CDK5 and its effect on the BIN1/c-MYC interaction in NSCLC cells. **a** The protein expression of BIN1 and CDK5 in NSCLC cell lines and embryo lung cells 2BS detected by western blotting. **b** The co-expression status of CDK5 and BIN1 in H460 and PC9 cells detected by immunofluorescence. **c** The efficiency of various siRNAs in suppressing the expression of CDK5. **d** The effect of CDK5 knockdown on the expression of CDK5, c-MYC and phosphorylated c-MYC at Ser-62 site in H460 cells. **e** The effect of CDK5 overexpression on the expression of CDK5, c-MYC and phosphorylated c-MYC at Ser-62 site in PC9 cells. **f** The effect of CDK5 on the interaction of BIN1/c-MYC in H460 and PC9 cells detected with Co-IP. Significant P-values marked by asterisk: *P < 0.05

To preliminarily clarify whether presence of CDK5 could act as tumor promoter by effecting the interaction of BIN1/c-MYC, we detected both CDK5 and BIN1 expression status in NSCLC cells. First, we used western blot to detect BIN1 expression in five NSCLC cells with high c-MYC expression (H460, H1299, H1975, PC9, H1792 and A549) and human embryo lung cell line 2BS. As shown in Fig. 1a, compared to 2BS cells, H1975, H1299 and A549 cells showed significantly low expression of BIN1 (P < 0.05) while PC9, H1792 and H460 cells showed a similar expression level (P > 0.05). Then, we evaluated CDK5 expression in these NSCLC cells and found H460, H1975, H1299, A549 cells showed high CDK5 expression (P < 0.05) while PC9 and H1792 cells showed low CDK5 expression (P > 0.05) (Fig. 1a). According to these results, PC9 and H460 cells were chosen for our further experiments.

**Fig. 2:**
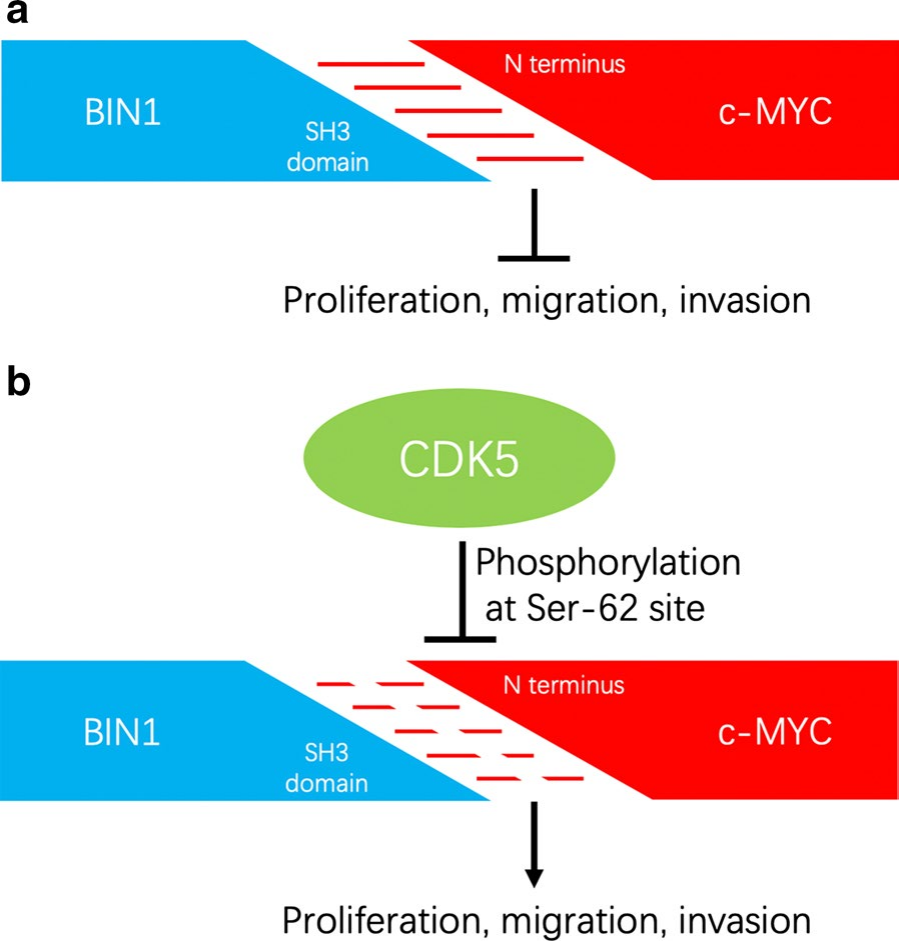
Schematic of the proposed effect of CDK5 on the interaction between BIN1 and c-MYC. **a** BIN1 could bind to the N terminus of c-MYC with its SH3 domain to inhibit the proliferation, migration and invasion ability of NSCLC cells. **b** CDK5 could induce the phosphorylation of c-MYC at Ser-62 site. Thus, the presence of CDK5 could block the interaction between BIN1 and c-MYC to neutralize the tumor-suppressing effect of BIN1

Aiming at confirming the potential of CDK5 in neutralizing BIN1/c-MYC interaction, we next determined the co-expression status of CDK5 and BIN1 in PC9 and H460 cells. The immunofluorescent results showed that CDK5 and BIN1 could be simultaneously observed in H460 cells, while only BIN1 could be observed in PC9 cells (Fig. 1b). Above results were consistent with western blotting. Then, the expression of CDK5 in H460 cells was silenced with siRNA to evaluate its effect on the BIN1/c-MYC interaction. As the Fig. 1c demonstrated, the siRNA-1 showed the most suppressing effect on CDK5 expression in H460 cells (P < 0.05) and it was used in the next experiments. After CDK5 knockdown, we detected the phosphorylation status of c-MYC on Ser-62 in H460 cells, and found it was significantly reduced (Fig. 1d). Meanwhile, we performed CDK5 overexpression in PC9 cells. After CDK5 overexpression, we detected the phosphorylation status of c-MYC at Ser-62 site in PC9 cells, and found it was significantly increased (Fig. 1e). Then, Co-IP assay results revealed that the interaction ability of BIN1 and c-MYC was increased after CDK5 knockdown (Fig. 1f). The results demonstrated that CDK5 knockdown could restore the tumor suppressing of BIN1 by inhibiting phosphorylation of c-MYC on Ser-62 (Fig. 1f). Likewise, the interaction between BIN1 and c-MYC was neutralized after CDK5 overexpression (Fig. 1f). Taken above, these results revealed that presence of CDK5 could deactivate the tumor suppressing effect of BIN1 by mediating phosphorylation of c-MYC on Ser-62 (Fig. 2).

### CDK5 promoted proliferation, invasion and migration ability of NSCLC cells by inhibiting the tumor suppressing effect of BIN1

**Fig. 3:**
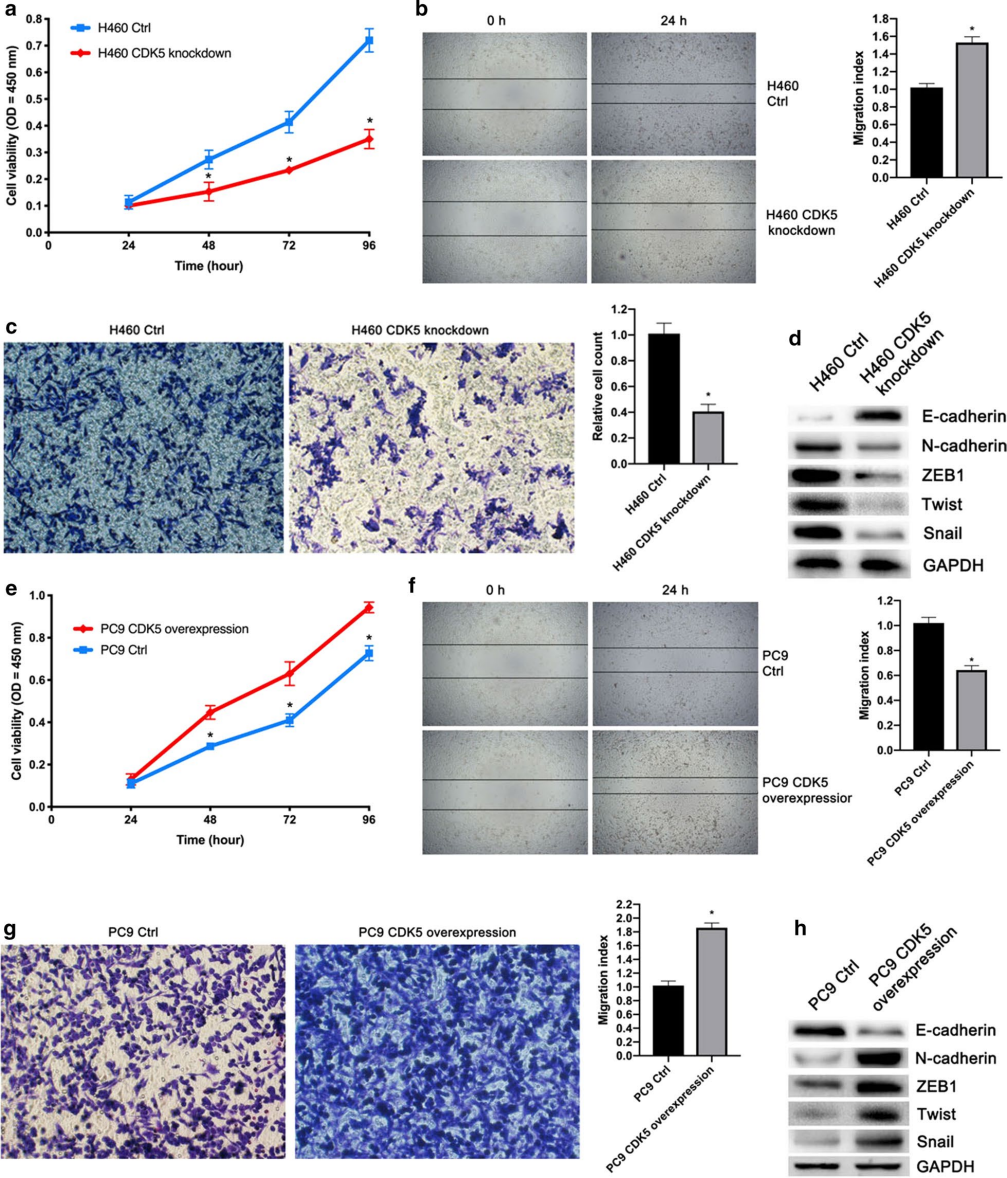
Effects of CDK5 on the malignant behaviors of NSCLC cells. **a** The effect of CDK5 knockdown on the growth ability of H460 cells. **b** The effect of CDK5 knockdown on the migration ability of H460 cells. **c** The effect of CDK5 knockdown on the invasion ability of H460 cells. **d** The effect of CDK5 knockdown on the expression of EMT-related proteins in H460 cells, GAPDH as control. **e** The effect of CDK5 overexpression on the growth ability of PC9 cells. **f** The effect of CDK5 overexpression on the migration ability of PC9 cells. **g** The effect of CDK5 overexpression on the invasion ability of PC9 cells. **h** The effect of CDK5 overexpression on the expression of EMT-related proteins in PC9 cells, GAPDH as control. Significant P-values marked by asterisk: *P < 0.05

As BIN1 has been proved to play a pivotal role in proliferation, invasion and migration abilities of cancer cells [21], we then detected whether CDK5 could affect these malignant behaviors by inhibiting the tumor suppressing effect of BIN1. Subsequently, CCK8 experiment was performed to further evaluate the effect of CDK5 overexpression on proliferation ability of H460 cells, and the results showed that the growth ability was notably suppressed after CDK5 knockdown (Fig. 3a). Meanwhile, the wound healing and transwell experiment were performed to determine the effect of CDK5 on migration and invasion ability of H460 cells. The results demonstrated that, after CDK5 knockdown, the migration and invasion ability of H460 cells were both reduced (Fig. 3b, c, P < 0.05). Since BIN1 could inhibit epithelial mesenchymal transition (EMT) [21], we detected the effect of CDK5 knockdown on the expression of EMT-related proteins in H460 cells. The results demonstrated that knockdown of CDK5 significantly upregulated the expression of E-cadherin while downregulated the expression of N-cadherin, ZEB1, Twist and Snail (Fig. 3d).

Parallelly, we detected the above malignant behaviors of PC9 cells after CDK5 overexpression. The results showed that the growth, migration and invasion ability were all increased in PC9 cells after CDK5 overexpression, compared to control cells (Fig. 3e–g, P < 0.05). Then, we detected the effect of CDK5 overexpression on the expression of EMT-related proteins in PC9 cells. The results demonstrated that overexpression of CDK5 could significantly downregulate the expression of E-cadherin and upregulate N-cadherin, ZEB1, Twist and Snail (Fig. 3h).

### Dinaciclib inhibited the expression of CDK5 to restore the BIN1/c-MYC interaction in vitro and suppressed tumor progression of NSCLC cells in vivo

**Fig. 4:**
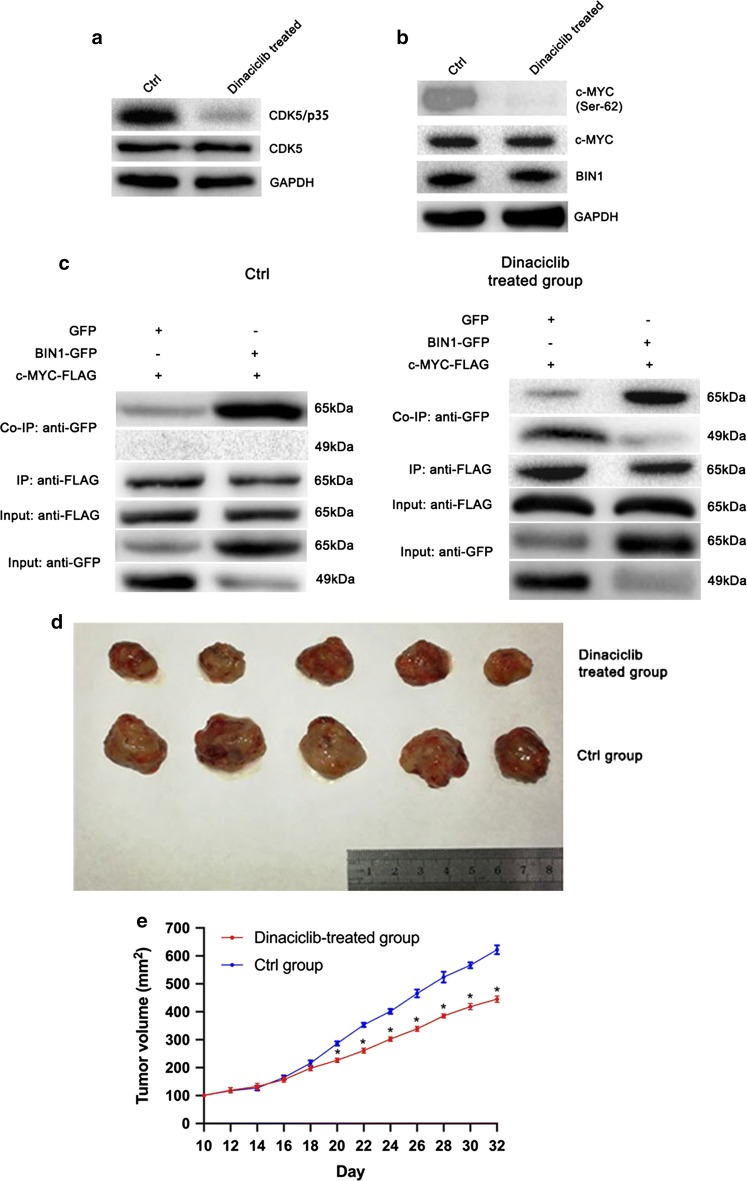
The effect of Dinaciclib treatment on H460 cells in vitro and in vivo. **a** The effects of Dinaciclib (concentration: 1 nM) on the expression of CDK5 and its activated form CDK5/p35 in vitro. **b** The effects of Dinaciclib (concentration: 1 nM) on the expression of phosphorylated c-MYC at Ser-62 site, c-MYC and BIN1. **c** The effects of Dinaciclib on the BIN1/c-MYC interaction. **d**, **e** The tumor volume of nude mice bearing with H460 cells. Significant P-values marked by asterisk: *P < 0.05

Then we determined whether CDK family inhibitor Dinaciclib had an effect on the BIN1/c-MYC interaction in NSCLC cells. Parry et al. demonstrated that Dinaciclib, a CDK family inhibitor without CDK5 specificity, showed the most efficient suppressing effect on the activity of CDK5 with little effect on other members of CDKs including CDK1, CDK2 and CDK9 at the concentration of 1 nM [22]. The results showed that the addition of Dinaciclib significantly decreased the expression of CDK5/p35 without any effect on the expression of CDK5 (Fig. 4a). This result indicated that Dinaciclib could suppress the activation of CDK5 at the concentration of 1 nM, since CDK5/p35 is the most important activated form of CDK5 [23]. Consequently, Dinaciclib suppressed the phosphorylation of c-MYC at Ser-62 site in H460 cells without having any effect on the expression of c-MYC and BIN1 (Fig. 4b). Thus, the blocking effect of CDK5 on the BIN1/c-MYC interaction could be neutralized by Dinaciclib in NSCLC cells (Fig. 4c).

To clarify the tumorigenesis mechanisms of CDK5, we constructed a tumor-bearing mouse model by injecting H460 cells into BALB/c nude mice. The tumor size was measured after modeling and randomly divided into 3 groups: Dinaciclib-treated group, placebo group, untreated group. Tumor volume in Dinaciclib-treated mice was significantly inhibited compared with placebo and untreated groups, indicating that loss of CDK5 could inhibit the tumorigenesis of NSCLC (Fig. 4d, e) (P < 0.05). In addition, we examined the phosphorylation status of c-MYC at Ser-62 site using western blotting. The results showed that the Ser-62 site of c-MYC was unphosphorylated in Dinaciclib-treated group, compared with that of control groups (Fig. 4f) (P < 0.05), suggesting that CDK5 inhibitor Dinaciclib suppressed proliferation via inactivation of c-MYC in vivo.

### Expression of CDK5 and BIN1 in tumor tissues was associated with prognosis of NSCLC patients

**Fig. 5:**
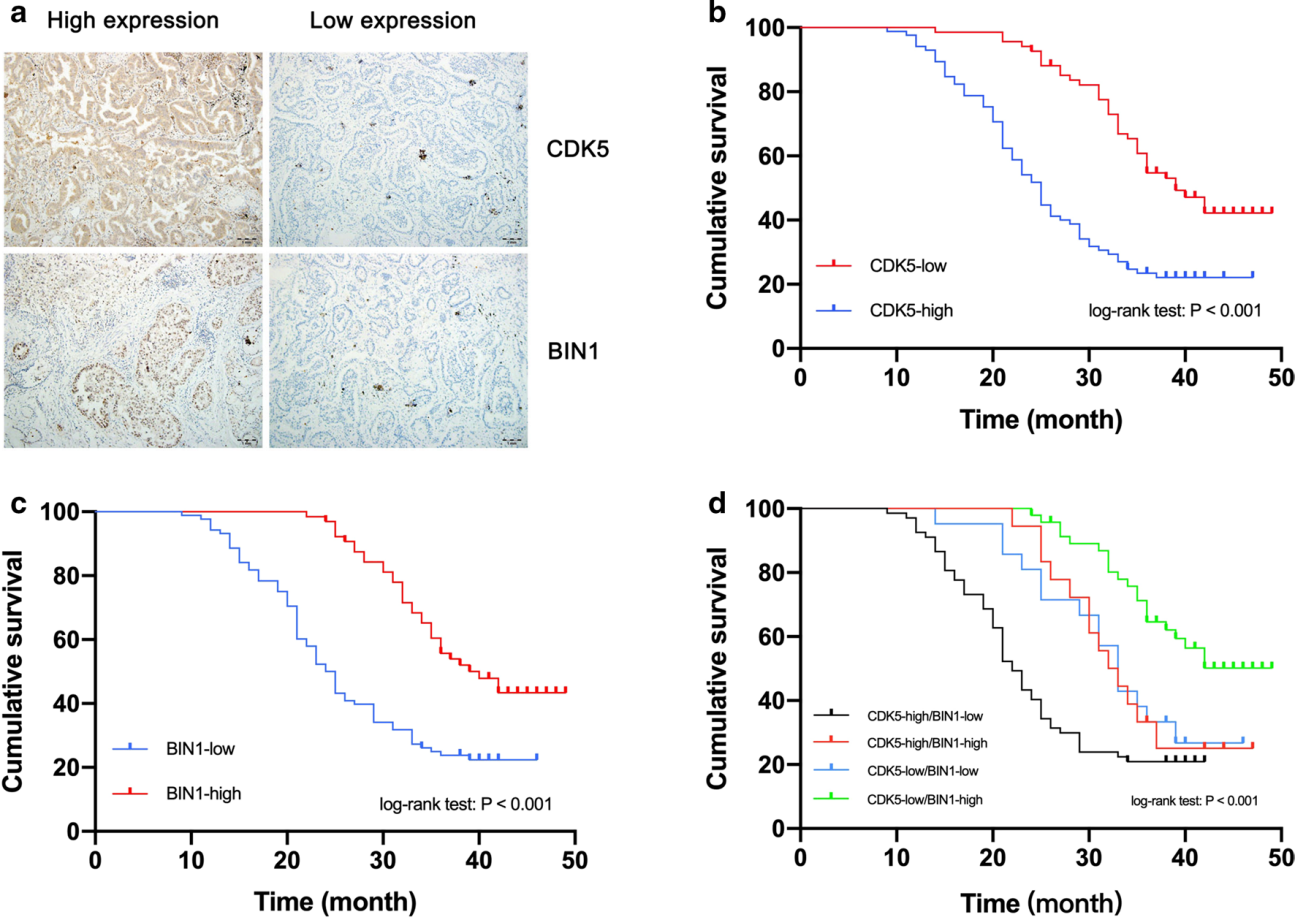
The role of CDK5 and BIN1 in predicting prognosis of the NSCLC patients. **a** The presentative expression of CDK5 and BIN1 in NSCLC tissues. **b** The correlation between CDK5 expression and overall postoperative survival of NSCLC patients. **c** The correlation between BIN1 expression and overall postoperative survival of NSCLC patients. **d** The correlation between co-expression status of BIN1 and CDK5 and overall postoperative survival of NSCLC patients

To reveal the expression pattern of BIN1 and CDK5, we detected their expressions in 153 NSCLC tissues by using IHC. As Fig. 5a showed, in the NSCLC tissues, the BIN1 staining, which occurred in cell nucleus, was found on few tumor cells. Meanwhile, the CDK5 staining occurring in cytoplasm could be found on most tumor cells. As Table 1 showed, CDK5 and BIN1 expression were both significantly related with the TNM stage, invasion range, and lymph node metastasis but not with gender and age (all P < 0.05). Kaplan–Meier analysis indicated that the overexpression of CDK5 was correlated with worse overall survival (OS) (Fig. 5b) (log-rank test: P < 0.001). The median OS for patients with high and low CDK5 expression were 25 and 39 months, respectively. These results indicated that the NSCLC patients with low CDK5 expression had better prognosis than those with high expression. In addition, high expression of BIN1 was correlated with better overall survival (Fig. 5c) (log-rank test: P < 0.001). The median OS for patients with high and low BIN1 expression were 40 and 24 months, respectively. which meant that NSCLC patients with high BIN1 expression had better prognosis than those with low expression. For further realizing the clinical significance of the co-expression status of CDK5 and BIN1, we divided the patients into four groups (CDK5-low/BIN1-high, CDK5-low/BIN1-low, CDK5-high/BIN1-high, and CDK5-high/BIN1-low) according to the expression status of CDK5 and BIN1, and then performed Kaplan–Meier analysis. The data showed that the OS of CDK5-low/BIN1-high group was shorter than patients in the other three groups (Fig. 5d) (log-rank test: P < 0.001). As Fig. 5d demonstrated, there was no significant difference in the OS between the CDK5-high/BIN1-low and CDK5-high/BIN1-high group while the OS of these two groups were both higher than CDK5-low/BIN1-low group. In conclusion, these results might provide some evidence that the expression of CDK5 and BIN1 could be potential biomarkers for predicting the prognosis of NSCLC patients.

**Table 1:** Associations of expression of CDK5 and BIN1 with patient clinical parameters of NSCLC patients

	n	Expression of CDK5 (n)		P	Expression of BIN1 (n)		P
		High	Low		High	Low	
Gender							
Male	103	61	42	0.190	43	60	0.791
Female	50	24	26		22	28	
Age							
≤ 60	109	62	47	0.604	46	63	0.912
> 60	44	23	21		19	25	
TNM stage							
I + II	86	33	53	< 0.001	47	39	0.001
III	67	52	15		18	49	
Invasion range							
T1 + T2	59	21	38	< 0.001	32	27	0.020
T3	94	64	30		33	61	
Lymph node metastasis							
Negative	75	27	48	< 0.001	43	32	< 0.001
Positive	78	58	20		22	56	

## Discussion

NSCLC is the one of the most common malignant tumor with unsatisfactory prognosis [24]. Aberrant activation of oncogenes is one of most important cancer hallmarks, which is closely related to worse prognosis [25–27]. c-MYC is known to be a frequent crucial oncoprotein in NSCLC, which is associated with poor prognosis and recurrence [28]. Therefore, interrupting of c-MYC regulatory units might be a potential alternative to indirectly inactivate c-MYC in tumors. BIN1 has been identified as a crucial tumor suppressor, which is important in numerous biological behaviors such as proliferation, apoptosis, DNA repair and immune escape [29]. Elliott et al. demonstrated that BIN1 could reverse c-MYC-mediated malignant transformation via directly interacting the N-terminus of c-MYC [11, 12]. However, aberrant phosphorylation of c-MYC at Ser-62 site could block the BIN1/c-MYC interaction [13]. In addition, CDK5 overexpression can directly phosphorylate c-MYC on Ser-62 [19]. Taken above, demonstrating whether CDK5 is involved in blocking BIN1/c-MYC interaction is beneficial for comprehending the biological function of CDK5 to establish novel therapeutic strategies in treating NSCLC.

In the present study, we evaluated the effect of CDK5 on BIN1/c-MYC interaction in NSCLC. CDK5, an aberrantly overexpressed protein in multiple cancers, could significantly promote proliferation, invasion and migration abilities of tumor cells [14, 15]. In hepatocellular carcinoma (HCC), CDK5 overexpression could promote proliferation of HCC cells to exert an oncogenic activity [30]. In prostate cancer, CDK5 increased cell growth ability in an androgen receptor (AR)-independent manner [31]. As for NSCLC, Wei et al. reported that CDK5 was significantly correlated with poor prognosis [32]. However, the concrete effects of CDK5 and related mechanisms in NSCLC still remain unclear. CDK5 phosphorylates a diverse list of substrates, implicating that it can regulate various cellular processes including the function of c-MYC, which is a crucial transcription factor in cancer [33, 34]. Aiming at further realizing the effect of CDK5 on BIN1/c-MYC interaction, we then detected its expression in NSCLC cell lines, and found that H460, H1299, H1975, and A549 cells showed high CDK5 expression, while PC9 and H1972 cells showed low CDK5 expression. Meanwhile, PC9, H460 and H1972 cells showed high BIN1 expression while H1299, H1975 and A549 cells showed low BIN1 expression. Thus, we chose PC9 and H460 cells for further studying the function of CDK5.

Knockdown of CDK5 expression in H460 cells significantly promoted the interaction of BIN1 and c-MYC. We found that knockdown of CDK5 could inhibit cell growth, invasion, migration and EMT ability of H460 cells. Meanwhile, we transfected CDK5 into PC9 cell which highly expressed BIN1, and the results showed that the growth, invasion, migration and EMT ability could be significantly enhanced by CDK5 overexpression. Furthermore, with Co-IP experiment, we found that CDK5 was correlated with the anergy of BIN1/c-MYC interaction, suggesting that CDK5 could neutralize the tumor-suppressing effect of BIN1 via inducing phosphorylation of c-MYC at Ser-62 site.

Due to the oncogenic function of CDK5, Dinaciclib, an effective inhibitor of CDK5, exerted potent tumor-suppressing effect in relapsed multiple myeloma and hepatocellular carcinoma [35, 36]. However, the function of Dinaciclib in NSCLC remains unclear. In present study, Dinaciclib could inhibit the growth, migration and invasion ability of NSCLC cells. We subsequently investigated the effect of Dinaciclib in vivo, and we found that mice injected with Dinaciclib-treated group developed significantly smaller tumors than mice injected with control group. These results provided some supporting evidence for its clinical implication in NSCLC.

## Conclusion

Taken together, to our best knowledge, despite of the studies on the carcinogenesis role of CDK5, this is the first study to analyze its effect on BIN1/c-MYC interaction. Knockdown of CDK5 significantly inhibited the phosphorylation of c-MYC at Ser-62 site to facilitate the interaction of BIN1 and c-MYC, and consequently suppressed the invasion and migration ability of NSCLC cells. High expression of CDK5 along with low expression of BIN1 could predict poor postoperative prognosis for NSCLC patients. Taken above, CDK5 might be used as a potential biomarker in predicting prognosis of NSCLC patients, and regarded as a novel target for anti-cancer therapies.

## Data Availability

We declared that materials described in the manuscript, including all relevant raw data, will be freely available to any scientist wishing to use them for non-commercial purposes, without breaching participant confidentiality.

## Abbreviations

NSCLC: non-small cell lung cancer
miRNA: microRNA
lncRNA: long non-coding RNA
CDK5: cyclin dependent kinase 5
BIN1: bridging integrator 1
EMT: epithelial mesenchymal transition
